# Metastatic neuroendocrine lesions in diffuse idiopathic pulmonary neuroendocrine cell hyperplasia (DIPNECH): a narrative review and case series

**DOI:** 10.1530/EO-25-0093

**Published:** 2026-05-27

**Authors:** David C Llewellyn, Saoirse Dolly, John K Ramage, Afsheen Wasif, Nabil Kibriya, Georgios K Dimitriadis, Amy M Llewellyn, Rajaventhan Srirajaskanthan

**Affiliations:** ^1^ Department of Endocrinology ASO/EASO COM, King’s College Hospital NHS Foundation Trust, London, UK; ^2^ Neuroendocrine Tumour Unit, King’s Health Partners ENETS Centre of Excellence, London, UK; ^3^ Faculty of Life Sciences and Medicine, King’s College London, London, UK; ^4^ Department of Histopathology, King’s College Hospital NHS Foundation Trust, London, UK; ^5^ Department of Radiology, King’s College Hospital, NHS Foundation Trust, London, UK; ^6^ King’s College Hospital NHS Foundation Trust, London, UK

**Keywords:** diffuse idiopathic pulmonary neuroendocrine cell hyperplasia, DIPNECH, metastasis

## Abstract

**Objective:**

Diffuse idiopathic pulmonary neuroendocrine cell hyperplasia (DIPNECH) is a rare entity characterised by the proliferation of neuroendocrine cells in the respiratory epithelium. DIPNECH is a pre-neoplastic condition that can progress to localised or metastatic neuroendocrine tumours (NETs). The understanding of DIPNECH remains limited, and although the European Neuroendocrine Society has recommendations for its management, there are currently no established guidelines.

**Method:**

This study involves a case series of three patients with DIPNECH progressing to metastasis, as well as a narrative review of previous cases screened from Medical Literature Analysis and Retrieval System Online (MEDLINE), Excerpta Medica dataBASE (EMBASE), Cochrane Central Register of Controlled Trials (CENTRAL) and SCOPUS databases.

**Results:**

In 40 documented cases of DIPNECH progression to metastasis, metastatic spread was more frequently observed in the thoracic lymph nodes, with a disproportionately high incidence of atypical carcinoid features on histological examination.

**Conclusion:**

Patients with DIPNECH are a heterogeneous group, and based on current data, it is difficult to confidently predict which are more likely to experience metastases. We provide recommendations for surveillance for a range of possible outcomes, including if there is DIPNECH without metastases, if there are no metastases but particularly large lesions and when metastases are present. Patients with DIPNECH-associated atypical neuroendocrine tumours, TNM stage > IIA, R1 resection margins and/or involvement of the visceral pleura ought to be monitored most closely. There are currently insufficient data to make meaningful recommendations regarding treatment options to prevent metastases.

**Learning points:**

- There are more published cases of metastases developing in the context of DIPNECH, suggesting that this is not an indolent condition in all cases.
- Thoracic lymph nodes tend to be the source of metastases, with a disproportionately high incidence of cases having atypical carcinoid features on histological examination.
- Patients with DIPNECH are a heterogeneous group, and based on current data, it is difficult to confidently predict which are more likely to experience metastases. Patients with DIPNECH and one or more of the following features: atypical neuroendocrine tumours, TNM stage > IIA, R1 resection margins and/or involvement of the visceral pleura, ought to be monitored as per ENETS and ESMO guidelines for atypical bronchial NETs. DIPNECH with typical bronchial NET should be followed as per ENETS and ESMO guidelines for typical bronchial NETs. Surveillance for nodules with no confirmed histology of DIPNECH should be followed as per the British Thoracic Society Guidelines.
- For patients with histologically proved DIPNECH but without visible lung neuroendocrine tumours, CT thorax surveillance scans for 1–2 years appear appropriate. If dominant lesions are >8 mm, due to increased chances of these sized lesions being malignant, it would be reasonable to undertake a baseline SSTR-PET (somatostatin receptor positron emission tomography) to confirm if any lesions are avid. If negative, the subsequent surveillance should be CT thorax, as long as there is no evidence of metastatic disease.
- If the baseline SSTR-PET scan demonstrates avidity and there is no discordant disease (i.e. all lesions identified on SSTR-PET are also visualised on CT), then CT surveillance alone is considered sufficient. However, small lesions <8 mm may be well below the spatial resolution of SSTR-PET and, therefore, should not be regarded as discordant disease. A repeat SSTR-PET scan is indicated only if there is evidence of lesion size progression or the emergence of new lesions on CT imaging.
- For patients with typical or atypical neuroendocrine tumours on a background of DIPNECH, as they appear to be more likely to metastasis, we advise following the recommendations made by the European Neuroendocrine Tumour Society for long-term follow-up post-excision.

## Introduction

Around 1% of normal respiratory epithelium is composed of neuroendocrine cells, which line the respiratory tract from the trachea down to the terminal bronchioles, and can be composed of solitary cells or clusters called neuroepithelial bodies (1, 2). Neuroendocrine cells are thought to be important in the fetal development of the lungs and are retained in smaller numbers in adults, continuing to secrete bioactive substances, such as calcitonin, calcitonin gene-related peptide, serotonin, chromogranin A and gastrin-related peptide (1, 2, 3, 4, 5). However, for reasons that remain unclear, in some patients, these cells can become hyperplastic; this phenomenon is termed diffuse idiopathic pulmonary neuroendocrine cell hyperplasia (DIPNECH).

DIPNECH is currently a purely histopathological diagnosis describing diffuse, multifocal neuroendocrine cell hyperplasia. DIPNECH may include tumourlets, which are clusters of neuroendocrine cells, <5 mm in size, which breach the basement membrane of the bronchiole, or tumours if ≥ 5 mm (6). DIPNECH is classified by WHO as a pre-neoplastic lesion which may develop into carcinoid tumours (typical or atypical) but it is not associated with high-grade neuroendocrine carcinomas. As previously noted by several authors, the definition of DIPNECH is somewhat controversial, with some arguing for a differentiation between patients with isolated histological features of DIPNECH and patients with histological features in association with classic symptomatology, sometimes referred to as DIPNECH syndrome (7, 8).

The pathology of DIPNECH is incompletely understood and appears to vary considerably between patients (9). The hyperplastic neuroendocrine cells are thought to release pro-inflammatory and pro-fibrotic bioactive substances, such as bombesin and serotonin. These substances lead to mild chronic inflammation, constrictive bronchiolitis and interstitial fibrosis, which results in abnormal lung function tests (9, 10, 11). However, these changes are not seen in every patient, and studies have previously demonstrated that only 44–53% of patients with a histological diagnosis of DIPNECH had features of chronic bronchiolitis and only 23–33% showed a bronchodilatory response to β agonists (11, 12). A recent large series reported a 46.3% improvement in symptoms for patients receiving β agonists +/− inhaled corticosteroids. Furthermore, 54.1% symptom improvement was noted in those receiving somatostatin analogues (13).

DIPNECH characteristically occurs in non-smokers aged 50–60 and is 10 times more prevalent in females than males. The cause of the strong female predominance is currently unknown. It was previously suggested that sex hormones may play a role in the development of DIPNECH; however, in a review of 19 patients, the hyperplastic neuroendocrine cells showed very low rates of immunoreactivity for oestrogen, progesterone and androgen receptors (7). We could find no evidence of autoimmunity being a factor. The most common presenting symptoms in patients with DIPNECH are a chronic non-productive cough, dyspnoea on exertion and wheezing (11, 12, 14, 15). The condition typically shows a slow progression, and patients have a good prognosis, with 83% alive 5 years after diagnosis (9, 12, 15, 16).

DIPNECH is a rare condition with non-specific symptoms and radiological findings. This results in frequent delays in diagnosis and high rates of misdiagnosis. Based on symptomology and pulmonary function tests, patients are often diagnosed with asthma or chronic obstructive pulmonary disease (9, 11, 17). Computerised tomography (CT) in patients with DIPNECH typically reveals a pattern of mosaic attenuation and airway thickening. Mosaic attenuation is a non-specific finding that can occur due to arterial obstruction in chronic thromboembolism, vasoconstriction in small airway diseases, or secondary to other causes of constrictive bronchiolitis, such as rheumatoid arthritis or Sjogren’s syndrome (10). In addition to these changes, some patients with DIPNECH have bilateral lung nodules which are round to ovoid and solid or ground glass with no calcification. The nodules are seen mostly along the small bronchovascular bundles in a centrilobular distribution, and usually less than 1 cm in diameter (9, 10, 16, 18, 19, 20). Further imaging can be used to review somatostatin receptor expression SSTR-PET (9). High somatostatin receptor expression is correlated with response to somatostatin analogues (SSAs); therefore, the imaging can help guide treatment options. However, there may be false negatives with these imaging modalities as the small tumourlets are below the spatial resolution of PET imaging (9).

Interestingly, there are significant differences between the neuroendocrine tumours arising in patients with a background of DIPNECH compared to patients who have isolated, sporadic neuroendocrine tumours with no DIPNECH (see Table 1). This indicates that, although both conditions involve pulmonary neuroendocrine hyperplasia, the underlying pathology is very different (7). In contrast to DIPNECH, sporadic neuroendocrine (carcinoid) tumours present in a younger age group. Patients with DIPNECH present with chronic cough, exertional dyspnoea and wheeze, compared to patients with peripheral sporadic neuroendocrine tumours who are usually asymptomatic and those with central neuroendocrine tumours who may present with a mild cough. There are case reports of patients with MEN-1 developing DIPNECH (21). Pulmonary function tests are typically normal in patients with sporadic neuroendocrine tumours, and CT scans reveal nodules with no mosaic attenuation or evidence of airway trapping (7). Histologically, patients with sporadic neuroendocrine tumours show higher mitotic counts and Ki-67 indices when compared to those with associated DIPNECH (15, 16). Finally, patients with sporadic neuroendocrine tumours have worse clinical outcomes than those with DIPNECH (15). However, it should be noted that it is difficult to accurately study the differences between these two groups of patients because to definitively categorise a patient as having a solitary neuroendocrine tumour that is not associated with DIPNECH, one would need to microscopically examine their whole lung parenchyma.

**Table 1:** Comparison of demographics, symptomology, radiological findings, histology and prognosis for patients with DIPNECH associated with NETs compared to sporadic NETs.

	DIPNECH-associated NETs	Sporadic NETs
Age	50–60+	Tend to be younger
Symptoms	May be asymptomatic, or cough, SOBOE, wheeze	Central lesions, mild cough
		Peripheral lesions, often asymptomatic
Lung function tests	Abnormal (obstructive picture with reduced FEV1)	Often normal
CT findings	Mosaic attenuation	Lung lesions
	Airway thickening	No attenuation or airway thickening
	+/−bilateral ovoid lung nodules	
	With no calcification	
Histology	Low grade	Tend to be higher grade
	Low Ki-67	
Five-year survival (not disease-specific)	80–90%	Overall 80–90%

Para-neoplastic syndromes, in the context of DIPNECH, are exceedingly rare. The reported incidence of para-neoplastic syndrome, primarily ectopic ACTH secretion, is around 1–5% of bronchial NETs (22, 23). Carcinoid syndrome is more common in bronchial NETs, accounting for between 4 and 30% of cases (24, 25).

DIPNECH is generally considered to be an indolent, slowly progressive condition; however, there are growing numbers of patients with metastases, even in the absence of an identifiable NET primary (26). Our knowledge of DIPNECH is in its infancy, and currently, there are no recognised guidelines for its management. We have performed a thorough narrative review of patients with DIPNECH and associated metastatic disease and present three patients treated at King’s College Hospital, who developed extrapulmonary metastases. By gathering and analysing data from existing cases, this study aims to provide physicians with evidence-based recommendations for surveillance frequency and appropriate imaging modalities. Furthermore, by identifying common characteristics among patients with metastases, we hope to contribute to a better understanding of the disease and potentially identify risk factors associated with the development of metastatic disease in DIPNECH.

## Method

Systematic searches of the following databases were performed: Medical Literature Analysis and Retrieval System Online (MEDLINE), Excerpta Medica dataBASE (EMBASE), Cochrane Central Register of Controlled Trials (CENTRAL) and SCOPUS databases. Our key MeSH (Medical Subject Headings) search term was the following: “DIPNECH” OR “diffuse idiopathic pulmonary neuroendocrine hyperplasia”. For each case of DIPNECH, we screened each paper to ensure no metastasis was missed.

Moreover, reference lists of selected articles and other literature sources were browsed to ensure a comprehensive literature search was completed. The search spanned from the beginning of databases to our last search in May 2025. Expert opinion manuscripts, letters to the editor, commentaries, conference papers, animal studies, meta-analyses and articles not in English were excluded. Data were only included on adults (18 years or older), men and non-pregnant women. No restrictions were made regarding where a study took place, the number of participants or the duration of follow-up.

### Case reports

We present three cases of patients diagnosed with DIPNECH who developed metastases, diagnosed and managed at King’s College Hospital, London.

#### Case 1

**Figure 1:**
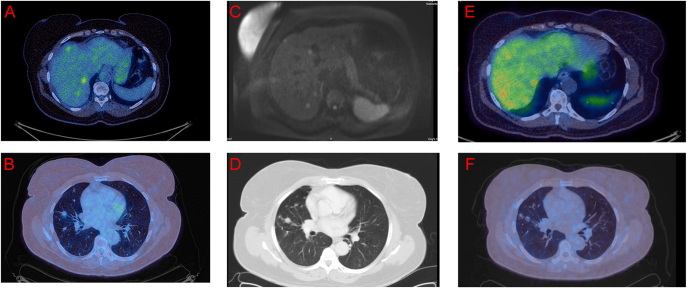
(A and B) ^18^F-FDG PET images from case 1, demonstrating low-grade avidity in the liver metastases (A) and dominant nodules (B). (C) MRI liver diffusion-weighted imaging identifies a number of small liver metastases. (D) CT thorax demonstrating a DIPNECH with dominant nodules. (E) ^68^Ga-DOTATATE PET imaging demonstrating avidity in some of the liver metastases. (F) ^68^Ga-DOTATATE PET imaging demonstrating low-grade avidity in the lung.

**Figure 2:**
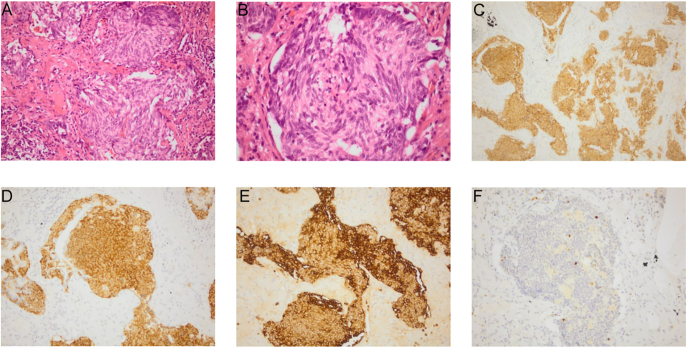
Histological findings from lung wedge resection, case 1. Multi-centric well-differentiated neuroendocrine tumour (typical carcinoid with multiple tumourlets). (A) Photomicrograph showing insular architecture with multiple tumourlets (x20 magnification). (B) High-power photomicrograph showing tumourlets composed of neuroendocrine cells with typical ‘salt and pepper’ chromatin pattern, no mitotic figures, no atypia and no necrosis (x40 magnification). (C) Immunohistochemistry for CD56, tumour showing strong, diffuse immunoreactivity (x20 magnification). (D) Immunohistochemistry for synaptophysin, tumour showing strong, diffuse immunoreactivity (x20 magnification). (E) Immunohistochemistry for chromogranin, tumour showing strong, diffuse immunoreactivity (x20 magnification). (F) Immunohistochemistry for Ki-67, highlighting <5% of tumour cells (x20 magnification).

A 65-year-old non-smoking Black British Caribbean female presented in 2014 with abdominal discomfort and a change in bowel habit but no respiratory symptoms. A CT scan showed thickening of the caecum and ^18^F-fluorodeoxyglucose (^18^F-FDG); positron emission tomography (PET) was performed as part of the staging process for expected colorectal cancer. It revealed liver lesions (see Fig. 1A). However, the subsequent colonoscopy was unremarkable with caecum biopsies of two pieces of tissue both returning normal. As part of the diagnostic workup for possible malignancy, the patient had imaging of the chest revealing multiple lung nodules with varying FDG avidity (see Fig. 1A and B). Later, in 2014, she underwent a video-assisted thoracic surgery (VATS) biopsy, showing DIPNECH with multiple neuroendocrine tumours, the largest of which measured 11 mm. Histology revealed marked desmoplasia surrounding the largest lesion, but no increase in mitotic activity (0 mitotic figures per 10HPF, Ki-67 < 5%) and no necrosis. Immunostaining confirmed that the lesions were neuroendocrine with positivity for CAM5.2, CD56, synaptophysin and chromogranin (Fig. 2). In view of history, radiological features and histopathological features, it was felt that there were no reasons for further staining to exclude other primary sites.

Over the next few years, imaging with ^18^F-FDG and SSTR-PET and MRI of the liver showed small lesions in all lobes of the lung, both peripheral and central, along with numerous liver metastases (see Fig. 1C and D). They remained mostly stable with only minimal metabolic progression and surveillance continued. No biopsy of the liver was performed as patient declined, but it was assumed to be secondary to DIPNECH based on imaging and the pattern of disease strongly suggesting that they were lung in origin. It is a recognised pattern of DIPNECH leading to metastasis, and there was no other clear source. Only two of the numerous lesions were DOTATATE-avid. In 2018, there was a mixed pattern of ^18^F-FDG/DOTATATE PET (^68^Ga-DOTATATE PET)-avid disease, with the lungs having increased ^18^F-FDG uptake compared to ^68^Ga-DOTATATE uptake and the liver having increased ^68^Ga-DOTATATE uptake compared to ^18^F-FDG (only low-grade avidity in the liver metastases, SUV max 4.4 and 6; see Fig. 1E and F). The lungs appeared stable, and while the liver MRI showed stable disease, the ^68^Ga-DOTATATE PET showed increased metabolic burden.

Due to the slow progression of liver metastases, lanreotide autogel 120 mg once monthly was commenced. This was subsequently stopped in 2021 as the patient developed pancreatitis, and it was felt that the medication may have contributed to her presentation. A ^68^Ga-DOTATATE PET at the end of 2021 showed minor progression, and so the decision was made to restart 90 mg of lanreotide autogel. Her ^18^F-FDG PET imaging of late 2022 showed a stable appearance of the multiple lung nodules and single liver nodule, which was an improvement compared to the 2021 imaging when there had been two liver lesions. Over the three years until April 2025, the SSTR-PET surveillance scans have shown stable appearance in the lungs and low-grade uptake in the liver. Her 2023 spirometry demonstrated normal lung function, with an FEV1 = 0.88, FVC = 1.14 and FV1/FVC = 77.2. She remains asymptomatic, with a performance status of 0. The radiological improvement from 2018, we feel, has demonstrated treatment success.

#### Case 2

A 78-year-old non-smoking Caucasian female with a background of eosinophilic asthma and atrial fibrillation. In 2021, she presented with dyspnoea despite optimal treatment for her eosinophilic asthma and a CT showed a possible inflammatory or neoplastic mass in the right lung which was avid on the ^18^F-FDG PET. Due to the severity of her asthma, it was decided not to perform a biopsy and proceed directly to lobectomy. Histological examination of the lobectomy specimen showed marked sub-pleural fibrosis and patchy mild chronic inflammation. There were foci of DIPNECH with no areas of necrosis, no increase in mitotic activity (0 mitotic figures per 10HPF), no breach of the mucosal basal lamina and no lesions larger than 5 mm. Local lymph nodes showed no evidence of nodal metastases.

In 2022, an ^18^F-FDG PET revealed stable appearances of the lung nodules. However, there were three new liver metastases, which were FDG-avid (SUVmax 6.3 in segment 3; no SUVmax available for the other two segments). One of the liver metastases was subcapsular and close to the heart. ^68^Ga-DOTATATE PET showed no avidity at any site of disease.

A liver core biopsy was obtained, showing a metastatic neuroendocrine tumour composed of small oval-shaped cells with dark, enlarged nuclei arranged in an anastomosing tubercular architecture. The mitotic rate was increased (3 mitotic figures per 10 HPF, Ki-67 20.8%), and there were two foci of necrosis. The immunohistochemistry showed that the cells were positive for CK7, MOC31, chromogranin, synaptophysin and TTF1.

In 2023, CT imaging showed progression of the nodal disease in the mediastinum and of the liver nodules, with the largest nodule in the lung being 2.2 cm and abutting the superior vena cava. The positive TTF1 staining from liver biopsy, along with possible natural progression of DIPNECH led the MDT to believe this was from the lung and not thymic in origin. No biopsy was taken. No nuclear medicine scans have been performed since 2022. Lung function tests showed an obstructive picture with an FEV1 of 0.59 (36%), FVC 1.95 (92%), FEV1/FVC ratio of 30%, with a peak PEF of 133 L/min. Treatment options have been discussed with the patient, and at the time of writing she has opted not to start any further therapy. She remains on a monoclonal antibody (benralizumab 30 mg injection) for her eosinophilic asthma, with stable symptoms.

#### Case 3

A 61-year-old non-smoking Black British Caribbean female with a history of hypertension, type 2 diabetes and childhood asthma. In 2021, she suffered from severe COVID pneumonitis, during which a CT chest incidentally revealed a small nodule in the right upper lobe. After her COVID-related lung changes improved, follow-up CT chest imaging identified multiple nodules in her lungs, with the largest measuring 17 mm in the lower left lobe. She had a persistent dry cough and breathlessness on exertion; however, these were attributed to severe COVID pneumonitis. No pulmonary function tests were performed.

To investigate further, an ^18^F-FDG PET CT was performed, which showed moderate metabolic activity in two lung nodules, one in the left lower lobe and the other in the right upper lobe. In addition, there were multiple lung nodules with no significant ^18^F-FDG uptake, and no nodal or extrathoracic lesions were detected.

**Figure 3:**
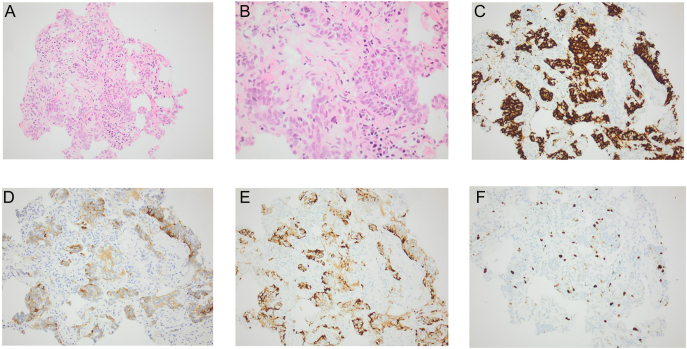
Histological findings from lung biopsy, case 3. (A) Photomicrograph showing multiple tumours composed of neuroendocrine cells (x20 magnification). (B) High-power photomicrograph showing neuroendocrine cells with typical ‘salt and pepper’ chromatin pattern, no mitotic figures, no atypia and no necrosis (x40 magnification). (C) Immunohistochemistry for CD56, neuroendocrine cells showing strong, diffuse immunoreactivity (x20 magnification). (D) Immunohistochemistry for synaptophysin, neuroendocrine cells showing immunoreactivity (x20 magnification). (E) Immunohistochemistry for chromogranin, neuroendocrine cells showing immunoreactivity (x20 magnification). (F) Immunohistochemistry for Ki-67, highlighting 2% of tumour cells (x20 magnification).

Subsequently, a core biopsy was conducted on the left lower lobe nodule, revealing classical features of DIPNECH with multiple small neuroendocrine tumours (Fig. 3). The biopsy displayed no mitotic figures, no necrosis and a Ki-67 index of 2%. In view of history, radiological features and histopathological features, it was felt there were no reasons for further staining to exclude other primary sites.

**Figure 4:**
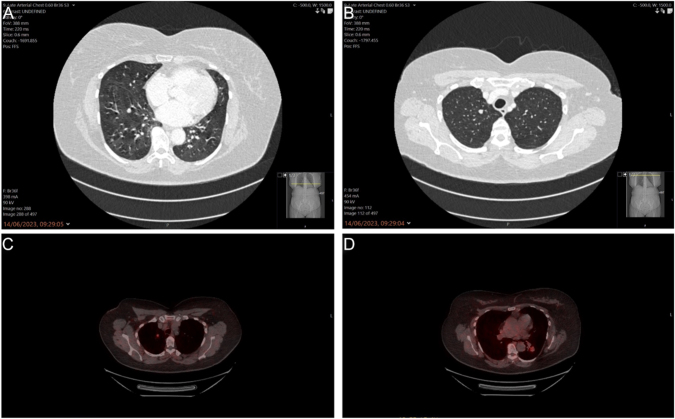
(A) Multiple small solid pulmonary nodules best demonstrated on MIP slab. Air trapping by constrictive bronchiolitis shows mosaic attenuation in affected areas. (B) Right upper lobe solid circumscribed nodule. Pulmonary nodules can affect all lobes with peripheral and lower zone predominance. (C) Right upper lobe ^68^Ga-DOTATATE-avid pulmonary nodule (somatostatin receptor positive). (D) Left lower lobe ^68^Ga-DOTATATE-avid pulmonary nodule.

To further investigate the neuroendocrine nature of the nodules, a ^68^Ga-DOTATATE PET was conducted later that year, confirming that the two previously identified lesions in the right upper lobe and left lower lobe were all ^68^Ga-DOTATATE-avid (Fig. 4). The scan also incidentally detected two foci of activity in the pancreas, but whether these were neuroendocrine lesions was unclear. Based on the diagnosis of DIPNECH, the patient was initiated on lanreotide autogel, 90 mg monthly. We excluded MEN-1 based on the imaging and no clinical or family history to suggest MEN-1, and it would be unexpected considering the patient’s age; hence, we did not do genetic screening.

To look more specifically at the pancreatic lesions, an MRI was performed which confirmed the presence of metastatic deposits in the tail of the pancreas and segment 7 of the liver. Her dose of lanreotide was increased to 120 mg monthly.

In 2022, the patient underwent regular surveillance CT imaging of the chest, abdomen and pelvis with additional pancreatic MRI scans. These scans revealed stable appearances of the lung and the 7 mm tail of pancreas lesions; however, a liver metastasis increased in size from 15 to 27 mm. No biopsy was taken as the patient declined. As with case 1, we assumed the lesions were secondary to metastatic spread from DIPNECH, based on imaging and the pattern of disease, strongly suggesting that they were lung in origin. It is a recognised pattern of DIPNECH leading to metastasis, and there was no other clear source. Following multi-disciplinary team discussion, the patient’s medication was changed to a mammalian target of rapamycin (mTOR) complex 1 inhibitor, namely everolimus 5 mg once daily. Unfortunately, as a side effect of everolimus, she developed pneumonitis within the first cycle and stopped treatment.

In 2023, repeat MRI scans of the liver revealed a further increase in the size of the metastases to 32 mm. The patient has been offered various alternative systemic therapies but has opted for ongoing surveillance imaging. The imaging performed on October 2024 showed the liver and lung lesions had minimal-to-no DOTATATE activity, suggesting that the use of peptide receptor radionucleotide therapy (PRRT) would be limited. In 2024, the patient was having symptoms of coughing and flushing, but no diarrhoea or wheezing. Performance status was 2, and the patient elected to continue lanreotide. At present, she is genuinely well, other than 2–3 kg of weight loss and gradual fatigue.

Cases 1 and 3 refused biopsy of the metastasis, but the imaging and pattern of disease strongly suggest that they originated from the lung. It is a recognised pattern of DIPNECH leading to metastasis, and there was no other clear source, which we are highlighting.

## Results and discussion

A summary of all previous reports of patients with metastatic neuroendocrine lesions associated with DIPNECH is shown in Supplementary Table 1 (see section on Supplementary materials given at the end of the article).

From a thorough review of the literature, the patient demographics of those with metastatic disease were indistinguishable from those described in general in patients with DIPNECH. Most patients were non-smoking females aged between 50 and 60. One study reported an association with raised BMI, hypertension and diabetes, suggesting that there may be a metabolic component (16). Some patients presented with classic respiratory symptoms suggestive of DIPNECH; however, there were several, including the patient presented in case 2, who were asymptomatic (11, 26, 27, 28, 29).

DIPNECH is a pre-invasive lesion with the potential to progress very slowly. Chung *et al.* described metastatic lesions developing 123 months after initial diagnosis of DIPNECH (19). Our case 1 took several years before metastases occurred. However, it is common for non-metastatic DIPNECH nodules to increase in size and not lead to metastasis. Little *et al.* showed that by 3.4 years from diagnosis, 67% of their 30 patients had an increase in size of the largest nodule, and 53% in size of their other nodules or new nodules forming (30). Almquist *et al.* found that 49% of their cohort had an increase in size or number of nodules, with a mean progression to carcinoid of 6 years (31). It is clear that clinicians cannot just assume that larger or new nodules in the lung are a sign that metastasis could develop. Multiple papers with a sufficiently sized cohort of patients have not seen any metastases, even if there are numerous patients with lesions growing in size (32, 33).

Metastases are most likely within the thoracic lymph nodes, and occur if the nodules have developed into typical or atypical carcinoid. Typically, around 50–70% of patients with DIPNECH also have carcinoid tumour within the histology (12, 30, 34). Around 50% of the time, histology showed these are typical carcinoid tumour, whereas only 2–22% have atypical carcinoid (19, 32, 35). Given the relative rarity of atypical carcinoids, our narrative review identified a noteworthy number of cases involving metastasis from atypical carcinoid tumours. Consequently, if histological analysis of a patient’s lung lesion indicates an atypical carcinoid, this should raise heightened concern for the potential development of future metastases.

Others have suggested features that were suggestive of poor prognosis and the development of metastatic disease were TNM stage > IIA, R1 resection margins and involvement of the visceral pleura (16, 26, 36). However, when comparing baseline and follow-up imaging for the patients with and without metastases, Chung *et al.* found that there were no features that could be used to predict the likelihood of future metastases (19).

It is also difficult to identify any histopathological features that may be pertinent in determining the metastatic potential of DIPNECH-associated neuroendocrine tumours as very few papers included a detailed histological description. Features such as mitotic rate, Ki-67 index or the presence of necrosis were frequently omitted. However, metastases did appear more frequent in patients with atypical neuroendocrine tumours compared to typical neuroendocrine tumours (16, 26). As mentioned previously, accurate histological characterisation is challenging as DIPNECH is a diffuse process which is usually diagnosed on small biopsies. It is possible that many more of the patients who eventually developed metastases had underlying atypical neuroendocrine tumours that were missed on the initial biopsy.

There is some evidence to suggest that peak flow tests (PFTs) worsen as pulmonary nodules enlarge and that PFTs could, therefore, be used as a simple, non-invasive follow-up technique (9). However, as noted previously, under the current definition some patients with DIPNECH have normal PFTs and Carr *et al.* found no correlation between decline in FEV1% and the number or size of nodules on HRCT scan (12). Based on our clinical experience and the cases reviewed, we would recommend careful long-term imaging follow-up.

Evaluating the efficacy of different medical treatments in patients with DIPNECH and designing appropriate treatment pathways is challenging as it is a rare, slow-progressing disease with heterogeneous symptomology and outcomes. Patients with DIPNECH are often prescribed oral or inhaled corticosteroids and short or long-acting beta agonists as a first-line agent to help with respiratory symptoms (9, 11, 37, 38). There have also been publications with small groups of patients showing symptomatic improvement with azithromycin, trialled due to its anti-inflammatory effect (39). Other approaches have focused on the aberrant activation of the mechanistic target of rapamycin (mTOR) pathway, which is known to be critical in the development of neuroendocrine neoplasms (7, 40). Studies into the use of sirolimus, or its derivative everolimus, in patients with DIPNECH have shown mixed results (41). Some cohorts have shown improvement or stabilisation of symptoms with radiological improvement; however, others have shown improvement in symptoms but radiological progression (11, 39, 40). Finally, somatostatin analogues (SSAs) have been reported to improve PFTs and symptoms and can help prevent progression to neuroendocrine tumours (20, 31, 42, 43). SSAs are widely used in the treatment of other neuroendocrine neoplasms and are thought to have both an anti-secretory and an anti-proliferative effect (11, 44). Currently, there is no strong evidence that SSAs or any of the other medical therapies prevent transformation to neuroendocrine tumours or development of metastatic malignancy (43).

Surgical resection of localised disease is widely recommended in the treatment of DIPNECH and in the study by Prieto *et al.*, 92% of patients underwent major pulmonary resections (16). Segmentectomy appears to offer the greatest prevention against tumour recurrence; however, DIPNECH can be very diffuse and, therefore, not all patients will be suitable for this type of surgery (30). Furthermore, as highlighted in the second case presented here, this type of surgery can have a significant impact on a patient’s performance status and quality of life. Before recommending surgical intervention, all cases should be discussed at a specialist multi-disciplinary team meeting to review a patient’s lung function tests, imaging, the severity of their symptoms and their personal preferences.

As highlighted by the findings of this narrative review, DIPNECH represents a heterogeneous condition and the underlying pathophysiology is poorly understood. Genetic sequencing would be useful to determine if there are any genetic or epigenetic factors which influence the likelihood of metastases. It would also be interesting to compare the sequencing results in patients with DIPNECH-associated neuroendocrine tumours and those with sporadic isolated neuroendocrine tumours to elucidate the differences between these two groups. Finally, more research is needed into the effects of environmental factors, such as smoking and exposure to pollution, on the development of respiratory symptoms and metastatic potential.

## Conclusion and recommendations

Metastatic spread from DIPNECH is uncommon; however, the number of reports is growing. The understanding of the condition is limited, and there is disagreement over its definition. Patients with DIPNECH are a heterogeneous group, and based on current data, it is difficult to confidently predict which are more likely to experience metastases.

On the basis of our clinical experience and review of the literature, we recommend that all patients with DIPNECH undergo lifelong imaging. There are no current recommendations for how often to survey patients with DIPNECH without neuroendocrine tumours. Considering DIPNECH lesions can be varied in the stage of progression, with innumerable lesions that cannot be biopsied, it is difficult to create surveillance guidelines. Plain CT thorax imaging yearly initially, and if stable, then extended to 2 yearly, appears appropriate (30, 32, 33). If dominant lesions are >8 mm, it would be reasonable to undertake a baseline SSTR-PET to confirm if any lesions are avid due to the risk of this size being malignant (45). If negative, the subsequent surveillance should be CT CAP, as long as there is no evidence of metastatic disease.

If the baseline SSTR-PET scan demonstrates avidity and there is no discordant disease (i.e. all lesions identified on SSTR-PET are also visualised on CT), then CT surveillance alone is considered sufficient. A repeat SSTR-PET scan is indicated only if there is evidence of lesion size progression or the emergence of new lesions on CT imaging (46).

For patients with typical or atypical neuroendocrine tumours on a background of DIPNECH, as they appear to be more likely to metastasis, we advise following the recommendations made by the European Neuroendocrine Tumour Society (ENETS) and European Society for Medical Oncology (ESMO) for long-term follow-up post-excision (46, 47). For patients with typical bronchial NETs, postoperative surveillance strategies vary according to tumour stage and resection status. In cases of complete resection (R0) with T1–T2 disease, CT imaging is recommended annually for the first two years, followed by imaging every three years thereafter. For patients with R0 resection and more advanced stages of the disease (T3–T4), CT surveillance should be conducted every six months during the first two years, and subsequently at intervals of every two to three years. In contrast, patients with microscopically incomplete resection (R1) require more intensive follow-up, consisting of CT imaging every three months for the first year, followed by six monthly scans for the next two years and annual imaging thereafter.

For atypical bronchial NETs, surveillance recommendations are similarly stratified by resection status and tumour stage. In patients with R0 resection and T1–T2 disease, an initial CT scan is advised at three months postoperatively, followed by annual imaging. For those with R0 resection and T3–T4 disease, CT imaging should be performed at three months, then every six months for five years and annually thereafter. In cases of R1 resection, a more intensive imaging schedule is recommended: an initial CT scan at three months, followed by imaging every three months for the first year, every six months for the subsequent two years and annually thereafter.

Patients with DIPNECH-associated atypical neuroendocrine tumours, TNM stage > IIA, R1 resection margins and/or involvement of the visceral pleura ought to be monitored most closely. Ideally, it would be good to understand the natural biology of DIPNECH in different patient groups, those without an associated bronchial NET and those with to determine the likelihood of development of progressive disease. However, due to the relatively low incidence of this condition, it would be difficult to recruit in a prospective manner.

There are currently insufficient data to make meaningful recommendations regarding treatment options to prevent metastases.

## Supplementary materials



## Declaration of interest

GKD is a full-time employee and shareholder of Eli Lilly and Company. All work reported in this manuscript was completed before transition to employment by Eli Lilly and Company. The authors declare that there is no conflict of interest that could be perceived as prejudicing the impartiality of this work.

## Funding

This work did not receive any specific grant from any funding agency in the public, commercial or not-for-profit sector.

## Author contribution statement

DCL contributed as the first author by researching previous cases and writing the paper. RS supervised and reviewed the paper. AW reviewed the histopathology images. AML assisted with writing the manuscript. GKD, SD and JKR reviewed and assisted with writing the manuscript. NK provided radiological imaging for the figures and, along with the other authors of this paper, contributed to reviewing and critiquing the analysis.

## Ethics

Written informed consent for publication of their clinical details and/or clinical images was obtained from the patients in the case series.
