# Supplementary material for: Metastatic neuroendocrine lesions in diffuse idiopathic pulmonary neuroendocrine cell hyperplasia (DIPNECH): a narrative review and case series
Source: Endocr Oncol. 2026 May 27;6(1):e250093. doi: 10.1530/EO-25-0093 (PMC13216853; doi:10.1530/EO-25-0093)
Supplement: Supplementary file 1 [file supplementary_materials.pdf]

Study year	Number DIPNECH (Number metastatic disease)	Demographics	Symptoms	Lung function tests	Radiological findings	Type and location of metastases	Treatment	Outcome
**Davies 2007**[1]	19 (1, 5.3%)	1 patient, unknown demographics	Asymptomatic	Unknown	Unknown	Atypical carcinoid to mediastinal lymph nodes Chemotherapy, unclear which type	Chemotherapy, unclear which type	Unknown
**Aubrey 2007**[2]	28 (4, 15.3%)	63yr M ex-smoker. 63yr and 54yr F never smoked. 1 other patient with unknown demographics	M-no symptoms 2 x F symptomatic. One other patient unknown if symptomatic	M-normal. 2xF had obstructive picture. Other patient unknown result	Unknown for all 4 patients	3 patients had a dominant carcinoid with lymph node metastases including one patient with N1 (stage IIA) disease and two patients with N2 (stage IIIA) disease. One other patient had metastases to the eye and bone.	Patient with metastases to eye and bone had SSAs and radiotherapy	Patients with lymph node only metastases did not progress. The patient with extrapulmonary metastases did not respond to treatment
**Lim 2010**[3]	2 (1, 50% - case reports)	72yr F ex-smoker	Dry cough, unknown duration	Unknown	FDG PET – multiple small (<5mm) lesions without uptake and a mild/moderate uptake of 18mm lesion in LLL	0.25mm metastatic typical carcinoid in the mediastinum	Lobectomy and mediastinal lymph node dissection	Unknown
**Irshad 2010**[4]	Case report	36yr white F non smoker	6 weeks of dry cough	"unremarkable"	CXR-extensive nodular infiltrates right lung. Low grade FGD uptake of nodules in right lung, no uptake in left lung.	Typical carcinoid tumour, vascular and nodal invasion but no extrapulmonary spread	Right pneumonectomy with radical hilar and mediastinal lymphadenectomy	One year later exercise capacity almost back to baseline
**Gorshtein**	11 (3,	3 white F 53-74	Unclear	Unclear	Unclear	Two patients had	SSA (unclear which	Still alive at end of follow

**2012**[5]	27.3%)	yr.				atypical carcinoid in the ipsilateral hilar nodes, one patient atypical carcinoid to adrenal gland	patients received lanreotide and octreotide). Disease stabilised.	up = 4.63 +/- 2.04 years
**Al-Ayoubi 2014**[6]	Case report	81yr F, smoker	Unknown	Mild obstructive disease with a predicted FEV1 of 66%.	Lesion in the right lower lobe but no extrapulmonary spread	Typical carcinoid tumour to the chest wall	No treatment for the DIPNECH	No recurrence or progression of DIPNECH
**Ofikwu 2015**[7]	Case report	46yr African American F non-smoker	Cough, dyspnoea, wheeze and occasional chest pain for 6-7years	FEV1 of 145L/min and 180L/min before and after salbutamol treatment	A PET scan showed uptake in bilateral lung nodules (largest 1.5cm) as well as the left hilar region	Carcinoid tumour with mediastinal metastasis	Bilateral VATS with resection of the 2cm right hilar lymph node, wedge resection of the middle lobe and excision of the right lower lobe	Mostly symptom free 2 years later
**Zagurovskaya 2017**[8]	Case report	51yr white F	Chronic cough	Unknown	2.1cm solitary pulmonary nodule medially located in the left lower lobe which was hypermetabolic	Atypical carcinoid with metastatic invasion of the left lower lobe bronchus, the adjacent left inferior pulmonary vein, local lymph node. 2 years later a 7mm breast metastasis, plus neuroendocrine cell brain metastasis was detected	Sandostatin injections following left lower lobectomy and partial mediastinal lymph node dissection. Whole brain radiation therapy and everolismus	Unknown
**Flint 2019**[9]	Case report	60sF non-smoker	Years of dry cough, 1 month nausea+ vomiting	Unknown	Severe diffuse mosaic attenuation with innumerable bilateral lung nodules, mostly along the small bronchovascular bundles. Largest was 16.3mm.	Atypical carcinoid R lower lobe lung. Metastases to liver.	Octreotide	Deteriorated with sepsis and transitioned to hospice care

					Abdominal MRI showed hepatic masses to both lobes			
**Samhouri 2020** [10]	44 (1, 2.3%)	Unknown	Unknown	Unknown	Unknown	Typical carcinoid (site unknown)	Everolimus with cabozantinib and a programmed death ligand-1 inhibitor	Symptoms stable, but radiological progression
**Little 2020**[11]	32 (2, 6.3%)	Unknown	Unknown	Unknown	Unknown	1 atypical carcinoid, 1 unknown, both metastasised to liver	Unknown	Unknown
**Sousa 2021**[12]	Case report	95F non smoker	Dry cough and exertional dyspnoea + peripheral oedema	Unknown	CT – bilateral nodules largest 9mm, bronchial thickening + bronchiectasis	Typical carcinoid tumour<br>Metastatic (cardiac)	Diuretics, but otherwise conservative management	Died 8 months after diagnosis
**Prieto 2021**[13]	25<br>(9, 36%)	Unknown	Unknown	Unknown	Mostly mosaic attenuation with multiple bilateral nodules	Mediastinal	Major pulmonary resection	pN2 involvement worsened prognosis
**Chung 2021**[14]	27<br>(3,11.1%)	3x F, 61-67yr non-smokers	1 cough,<br>2 incidental findings	FEV1<br>78-80% predicted		All atypical carcinoids<br>1 nodal<br>2 extrapulmonary spread to liver and bone.	Unknown	Median follow up of 67 months showed new lymphatic spread and increase in nodule size and number of nodules
**Hayes 2022**[15]	61 (7, 11.5%)	6x F<br>1 x M.<br>Majority non-smokers	Mostly asymptomatic	Unknown	4/5 pts with TNM stage were $\geq$IIA	1 hilar<br>6 extrapulmonary	6 had chemotherapy<br>1 everolimus	4 of 7 died

Supplementary Table 1: Summary of all previous reports of patients with metastatic neuroendocrine lesions associated with DIPNECH

Abbreviations: DIPNECH=diffuse idiopathic pulmonary neuroendocrine cell hyperplasia, F=female, M-male, yr= years old, FEV1= forced expiratory volume/1 second, fluorodeoxyglucose-18 positron emission tomography (FDG PET), mm=millimetres, cm = centimetres, LLL= left

lower lobe, CXR = chest xray, FDG = fluorodeoxyglucose-18, MRI = magnetic resonance imaging, CT = computerised tomography, TNM= tumour, lymph node and metastasis, SSA= somatostatin analogue, VATS = video assisted thoracoscopic surgery, pts= patients

References:

1. *Davies SJ, Gosney JR, Hansell DM, et al. Diffuse idiopathic pulmonary neuroendocrine cell hyperplasia: an under-recognised spectrum of disease. Thorax. 2007;62(3):248-252. doi:10.1136/thx.2006.063065*

2. *Aubry MC, Thomas CF, Jett JR, Swensen SJ, Myers JL. Significance of Multiple Carcinoid Tumors and Tumorlets in Surgical Lung Specimens. Chest. 2007;131(6):1635-1643. doi:10.1378/chest.06-2788*

3. *Lim C, Stanford D, Young I, McCaughan B, Cooper W. Diffuse idiopathic pulmonary neuroendocrine cell hyperplasia: a report of two cases. Pathol Int. 2010;60(7):538-541. doi:10.1111/j.1440-1827.2010.02552.x*

4. *Irshad S, McLean E, Rankin S, et al. Unilateral diffuse idiopathic pulmonary neuroendocrine cell hyperplasia and multiple carcinoids treated with surgical resection. J Thorac Oncol. 2010;5(6):921-923. doi:10.1097/JTO.0b013e3181db6ddd*

5. *Gorshtein A, Gross DJ, Barak D, et al. Diffuse idiopathic pulmonary neuroendocrine cell hyperplasia and the associated lung neuroendocrine tumors: clinical experience with a rare entity. Cancer. 2012;118(3):612-619. doi:10.1002/cncr.26200*

6. *Al-Ayoubi AM, Ralston JS, Richardson SR, Denlinger CE. Diffuse pulmonary neuroendocrine cell hyperplasia involving the chest wall. Ann Thorac Surg. 2014;97(1):333-335. doi:10.1016/j.athoracsur.2013.04.139*

7. *Ofikwu G, Mani VR, Rajabalan A, Adu A, Ahmed L, Vega D. A Rare Case of Diffuse Idiopathic Pulmonary Neuroendocrine Cell Hyperplasia. Case Rep Surg. 2015;2015:1-5. doi:10.1155/2015/318175*

8. *Zagurovskaya M, Tran-Harding K, Gibbs R. Primary lung carcinoid metastatic to the breast. Radiol Case Rep. 2017;12(2):223-228. doi:10.1016/J.RADCR.2017.02.003*

9. Flint K, Ye C, Henry TL. Diffuse Idiopathic Pulmonary Neuroendocrine Cell Hyperplasia (DIPNECH) with liver metastases. BMJ Case Rep. 2019;12(6). doi:10.1136/BCR-2018-228536

10. Samhouri BF, Azadeh N, Halfdanarson TR, Yi ES, Ryu JH. Constrictive bronchiolitis in diffuse idiopathic pulmonary neuroendocrine cell hyperplasia. ERJ Open Res. 2020;6(4):1-10. doi:10.1183/23120541.00527-2020

11. Little BP, Junn JC, Zheng KS, et al. Diffuse Idiopathic Pulmonary Neuroendocrine Cell Hyperplasia: Imaging and Clinical Features of a Frequently Delayed Diagnosis. American Journal of Roentgenology. 2020;215(6):1312-1320. doi:10.2214/AJR.19.22628

12. Sousa D, Rocha F, Baptista B, Horta AB. Diffuse Idiopathic Pulmonary Neuroendocrine Cell Hyperplasia With Progression to Neuroendocrine Tumor. Cureus. Published online February 13, 2021. doi:10.7759/CUREUS.13297

13. Prieto M, Chassagnon G, Lupo A, et al. Lung carcinoid tumors with Diffuse Idiopathic Pulmonary NeuroEndocrine Cell Hyperplasia (DIPNECH) exhibit pejorative pathological features. Lung Cancer. 2021;156:117-121. doi:10.1016/J.LUNGCAN.2021.04.024

14. Chung C, Bommart S, Marchand-Adam S, et al. Long-Term Imaging Follow-Up in DIPNECH: Multicenter Experience. J Clin Med. 2021;10(13). doi:10.3390/jcm10132950

15. Hayes AR, Luong TV, Banks J, et al. Diffuse idiopathic pulmonary neuroendocrine cell hyperplasia (DIPNECH): Prevalence, clinicopathological characteristics and survival outcome in a cohort of 311 patients with well-differentiated lung neuroendocrine tumours. J Neuroendocrinol. 2022;34(10). doi:10.1111/JNE.13184
